# Impact of Cumulative Environmental and Dietary Xenobiotics on Human Microbiota: Risk Assessment for One Health

**DOI:** 10.3390/jox12010006

**Published:** 2022-03-17

**Authors:** Pilar Ortiz, Alfonso Torres-Sánchez, Ana López-Moreno, Klara Cerk, Ángel Ruiz-Moreno, Mercedes Monteoliva-Sánchez, Antonis Ampatzoglou, Margarita Aguilera, Agnieszka Gruszecka-Kosowska

**Affiliations:** 1 Department of Microbiology, Faculty of Pharmacy, University of Granada, Campus of Cartuja, 18071 Granada, Spain; piortiz@ugr.es (P.O.); alfons_ats@hotmail.com (A.T.-S.); alopezm@ugr.es (A.L.-M.); klaracerk@ugr.es (K.C.); angel173@correo.ugr.es (Á.R.-M.); mmonteol@ugr.es (M.M.-S.); ampatzoglou@ugr.es (A.A.); 2 Institute of Nutrition and Food Technology “José Mataix” (UGR-INYTA), Centre of Biomedical Research, University of Granada, 18016 Granada, Spain; 3 IBS (Instituto de Investigación Biosanitaria ibs.), 18012 Granada, Spain; 4 Department of Environmental Protection, Faculty of Geology, Geophysics and Environmental Protection, AGH University of Science and Technology, Al. Mickiewicza 30, 30-059 Krakow, Poland

**Keywords:** xenobiotic, risk assessment, farm to fork strategy, ADME approach, microbiome

## Abstract

Chemical risk assessment in the context of the risk analysis framework was initially designed to evaluate the impact of hazardous substances or xenobiotics on human health. As the need of multiple stressors assessment was revealed to be more reliable regarding the occurrence and severity of the adverse effects in the exposed organisms, the cumulative risk assessment started to be the recommended approach. As toxicant mixtures and their “cocktail effects” are considered to be main hazards, the most important exposure for these xenobiotics would be of dietary and environmental origin. In fact, even a more holistic prism should currently be considered. In this sense, the definition of One Health refers to simultaneous actions for improving human, animal, and environmental health through transdisciplinary cooperation. Global policies necessitate going beyond the classical risk assessment for guaranteeing human health through actions and implementation of the One Health approach. In this context, a new perspective is proposed for the integration of microbiome biomarkers and next generation probiotics potentially impacting and modulating not only human health, but plant, animal health, and the environment.

## 1. Introduction

**Figure 1:**
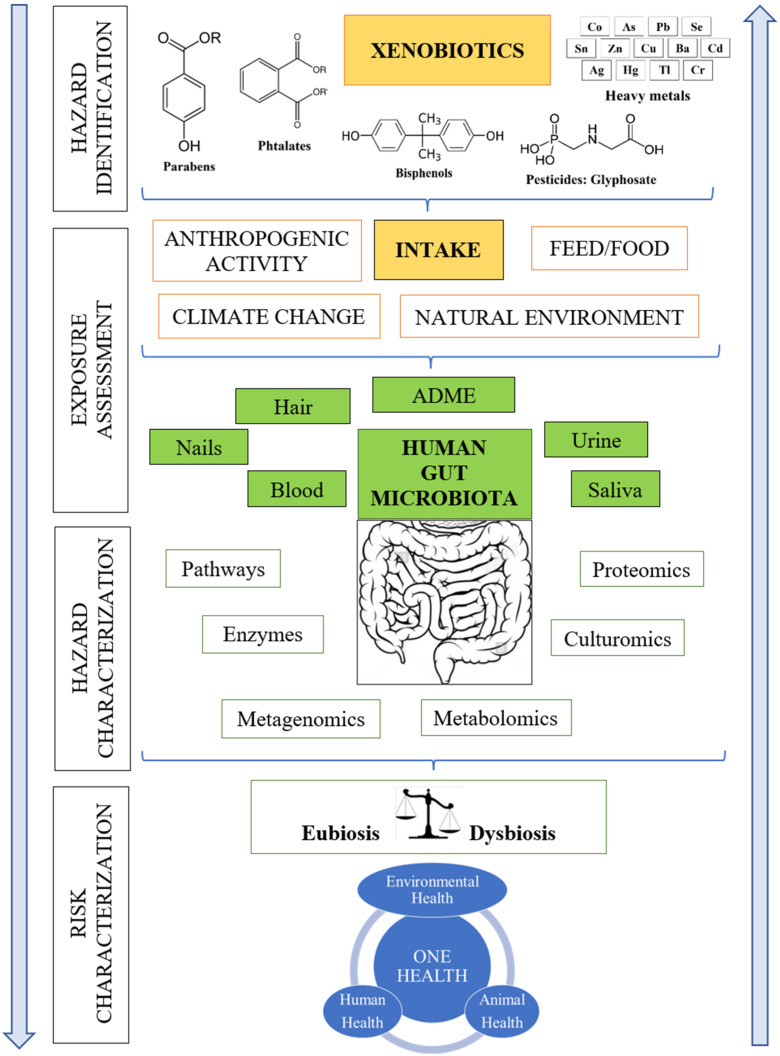
Risk assessment of xenobiotics in an accordance with the One Health approach.

Ongoing pollution due to anthropogenic activities poses a significant threat to the environment and health of its inhabitants. Until recently, human health was the primary focus of risk assessment in the context of the risk analysis framework. However, international policies [1] are starting to reflect research results revealing that humans are not a separate but an integral cog in the complex machine of the Earth’s ecosystem. Since the interdependencies between human, animal and plant health and the environment were realised, health started to be regarded through the prism of an integrated approach that is being called One Health. The definition of One Health refers to the holistic approach for simultaneously improving human, animal, and environmental health through transdisciplinary cooperation [2]. In this context, the human gut microbiome can be also considered as an important element of the next generation risk assessment in support of this One Health approach (Figure 1).

Anthropogenic activities, such as industrial processes, urbanization, waste disposal, agriculture, breeding, etc., introduce directly to the ecosystem various compounds, with the important common feature of being foreign to the biological system, and, as such, may cause unprecedented adverse effects in the ecosystem. These foreign to the body or the biological system substances are referred as xenobiotics [3]. Currently, the most investigated groups of xenobiotics, i.e., pesticides, preservatives, plasticizers, personal care products, dyes, and pigments, have been found in the following products: plant control constituents, drugs, pesticides, cosmetics, flavourings, fragrances, food additives, industrial chemicals, and environmental pollutants [4]. On the other hand, these contaminants may also enter the ecosystem indirectly due to instability of the environment caused by environmental degradation (such as deforestation) and climate change. Moreover, these xenobiotics do not always remain in the same chemical form but can be transformed during chemical, physical, and biological processes [5].

Xenobiotic dietary exposure is a global health concern nowadays. As dietary intake is the most important exposure pathway related to the xenobiotic intake, the quality and quantity of food consumed is of particular interest among the research community.

Xenobiotics may alter the microbiota composition, leading to a state of dysbiosis, which is linked to multiple diseases and adverse health outcomes, including increased toxicity of some xenobiotics. Toxicomicrobiomics studied the mutual influences and alterations between the ever-changing microbiome cloud and xenobiotics of various origins, with emphasis on their fate and toxicity [6,7]. These bidirectional interactions could be diverse: (1) Individual gut microbiome (human/animals) might be negatively affected by several contaminants or xenobiotics with pathophysiologic impact through triggering microbial composition disequilibrium; (2) Gut microbiota could protect against the carcinogenic and genotoxic substances by degrading or biotransforming them to less toxic compounds or facilitating their excretion; (3) Gut microbiota may also detoxify xenobiotics, for example, into genotoxins, or may reverse the detoxification implied by host metabolism; (4) Gut microbiota is capable of transforming xenobiotics towards lower toxic and mutagenic substances, thus it may be able to lessen the chances of dysbiosis effects.

However, dietary contaminants and hazardous substances present in environmental compartments may also significantly affect human health. Remarkably, regarding xenobiotic metabolism, participation of human gut microbiota might mediate long term physiological impact, affecting the balance between eubiosis and dysbiosis. However, to understand the key mechanisms a multidisciplinary approach is needed. Xenobiotics that alter the gut microbial composition and metabolism is categorized into a subgroup termed “microbiota disrupting chemicals” (MDCs) [8]. These MDCs might have the ability to promote changes in the microbiota that have been associated with intestinal, hormonal, and chronic or long-term systemic diseases in the host.

A collection of MDCs and their effects on the microbiota has widely explained by several authors: bisphenol [9,10]; phthalates, such as diethylhexylphthalate [11,12]; heavy metals. Many heavy metals have shown disrupting properties, and human exposure occurs through diet and water, inhalation of polluted air, smoking, and dermal absorption [13,14,15]. Triclosan and parabens: Triclosan is a well-known preservative [16]. Parabens are widely used as preservatives in cosmetics, personal care products, drugs, and foods [17]. Polybrominated diphenyl ethers (PBDEs) are environmentally persistent chemicals widely used as flame retardants [18] that have been shown to alter microbiota [19]. Pesticides have been shown to have important implications for environmental, animal, and human health [20]. Glyphosate and chlorpyrifos exposure occur mainly through diet and drinking water [21]. They have been reported to interfere with gut microbial communities and enteroendocrine cells [22,23]. Diazinon, an organophosphate pesticide with estrogenic activity [24], has shown to alter the structure of the gut microbiome community, functional metagenome, and associated metabolic profiles in a sex-related manner in murine models [25]. Organochlorine pesticides with endocrine disrupting capacity have also been associated with alterations in gut microbiota [26,27]. Antibiotics are also MDCs of concern, especially because of their contribution to antimicrobial resistance, a critical One Health issue [28]. Directly and indirectly (e.g., via reducing short-chain fatty acid production), they can have profound short and long-term negative effects on the gut microbiome, including altering its composition and opening a niche for pathogens [29]. Additionally, due to ingestion exposure to antibiotic-resistant microorganisms and given its high microbial density, the gut microbiome is emerging as a reservoir for antibiotic resistance genes and their horizontal transfer to commensals and, importantly, pathogens [30,31].

The cumulative exposure to xenobiotics and overall outcome could hugely impact health research. Therefore, interaction among distinct scientific disciplines as microbiology, nutrition, toxicology, environmental protection, and both personalised medicine and nutrition are needed. Moreover, multiple technologies, communities and professional domains should converge in order to obtain relevant outcomes. Research in risk assessment, including microbiota, metabolome, and omics technologies, favours new progress to evaluate the factors and substances that affect human health. Studies employing holistic analysis of human microbiota as metabolic node for health impact is the first step of this perspective goal.

The main objective of this work is to pay attention to the upmost scientific information about the interaction between the human microbiota and exposure to xenobiotics in the diet and environment.

## 2. Key Challenges of Gut Microbial Metabolism Research

Regarding the implementation of current EU policies, emissions of hazardous substances should be limited and controlled [32]. However, even so, xenobiotics already present in the environment cause a major problem, as demonstrated by the fact that even extensive human biomonitoring studies do not approach, adequately, the real body burden scenario [33]. Xenobiotics can be found everywhere in daily life, from food, cosmetics, and homecare products to prescription and over-the-counter drugs, gasoline, alcoholic beverages, and paper receipts. Thus, preventing long-term exposure to these harmful substances and mitigating their impacts become health and environmental priorities.

Interestingly, despite food being the source of energy and nutrients for the human body to grow, develop, and perform everyday activities, it may also contain xenobiotic substances. The rising of the global population results in higher food demand and consequently necessitates incremental increases in food resources [34]. Developed countries have incorporated much more processed foods and artificial products into the diet of their populations to keep up with the rapid pace of modern lifestyles. Cumulative exposure to contaminants and the physiological impact and mechanisms are poorly investigated [35]. Importantly, long-term exposure contributes to host gut microbiome dysbiosis [36], which previously had only been associated with changes in diet or antibiotic use [37,38]. Moreover, dysbiosis associated with xenobiotic exposure varies from person to person, depends on life stage and, overall, appears to be a strongly personalised effect [39].

As xenobiotic mixtures are increasingly ubiquitous in the environment nowadays [40], the question of how much the environment is polluted does not suffice anymore. The key question raised recently by the research community as well as by key environmental and health authorities, organisations, and agencies, such as EFSA, WHO, EEA, USEPA, etc., is what adverse health effects are caused by everyday contact with xenobiotics. In this context, the risk assessment consists of four crucial parts. The first step is hazard identification, followed by exposure assessment, hazard characterisation, and, finally, risk characterization (Figure 1). Regarding xenobiotics, the following challenges can be observed. Firstly, exact concentrations of defined xenobiotics are needed for reliable risk assessment. It means that specific xenobiotics entering the body via ingestion of food and via other exposure routes (e.g., inhalation or skin contact) facilitated by environmental pollution must be identified and their concentrations in foods/environment be elucidated. Secondly, xenobiotics may not remain in the same chemical form, but they may be transformed in other chemicals less or more toxic comparing with the parent compound. Moreover, the total concentration of xenobiotics does not always raise concerns, as only the bio-accessible proportions are responsible for adverse health effects. Additionally, the bio-accessibility of each xenobiotic is regulated by complex ADME processes (absorption, distribution, metabolism, excretion) taking place in living organisms.

Xenobiotic ADME processes involve genes, enzymes, and pathways not only of human origin, but also from the human microbiota, which has started to be investigated thoroughly in latest years. The current knowledge has shown that innovative microbiota biocomponents and functional analyses could contribute to increase the metabolites, analytes and enzymatic repertoire beyond the microbial taxonomic principal components analyses that were widely used in studies of the microbiome and toxicant exposures [41]. Recent research advancements have focused on molecular data for involving microbiome biomarkers in the prevention of diseases and dysfunctions triggered by xenobiotic exposures [42,43]. Integrative omics data and technologies [44,45] became essential tools for determining the holistic impact of microbiome on health-diseases balance (Figure 1). These findings strengthen the rationale for using a homologous model for toxicology. To study effects of exposures on the human microbiome in vitro systems may be preferable [46]. Moreover, the microbiome seems be particularly sensitive to xenobiotic influence during key life stages [47].

Personalized nutrition treatments based on next generation probiotics able to detoxify xenobiotics is envisaged as closing the circle of promoting the One Health approach [48]. Moreover, beneficial microbes isolated from the human gut microbiota could be further proposed for being used as plant probiotics, animal probiotics, and, even for bioremediation, having a potential positive modulation capacity in the context of One Health. The results of specific microbiota metabolizing particular xenobiotics and especially those used in bioremediation should be accompanied in parallel by environmental biosafety assessments [49].

Accordingly, as there is a strong drive-in international policy for next generation (non-animal) testing methods the human gut microbiota may become a plausible substitute to anticipate assessments and avoiding animal testing, when possible [50]. Specific consortia of human gut microbiota might be used in research studies to define the related ADME processes in cellular, organ-on-a-chip or organoids models [51] for analysing the fate of xenobiotics and inducting the effects in health and environment, such as for BPA biodegradation [52]. This contrasts with toxicological studies on animals, which are commonly used for environmental risk assessments performed up to now. Thus, the human gut microbiota approach seems to more relevant as results from traditional toxicology tests are performed on animals and in terms of acute exposure (short time, high doses) and, thus, must be extrapolated from animals to humans, as well as from high to low doses and long-term exposure that is specific for environmental exposure. Additionally, research determining the individual exposure levels of the human population depending on the composition of the intestinal microbiota may be performed as differences among individuals in the population are observed. The interindividual variations can be related to dietary habits, level of pollution, and ethnical and geographical differences [53].

According to Humboldt-Dachroeden et al. [54], One Health approach has the potential to become a comprehensive research field, however up to now environmental perspective of this approach is stunted in comparison for instance with epidemiological, microbiological, or public health perspectives. Environmental issues have gained even greater interest in terms of climate change and related adverse effects. Climate change disturbs the ecological and environmental integrity of living systems by causing lifecycle changes that at the end adversely affect among others food security and food safety, as well as related responses at local, regional, and global level [55]. In accordance with food security and food safety, European Union has launched the Farm to Fork Strategy, being the central part of the European Green Deal, for making food systems fair, healthy, and environmentally friendly, in one word—sustainable [56]. Food production is inseparably related with soil, thus in accordance with Green Deal Targets, European Commission has launched Mission Soils for achieving soil relevant targets in the perspective of 2030. Among Mission Soils’ targets related with the related with the One Health are following: reducing overall use and risk of chemical pesticides by 50%, reduce fertilizer us by at least 20%, reduce nutrient loses by at least 50%, reduce microplastic release into the environment by 30%, reduce the greenhouse gas emissions by at least 55% compared to 1990 levels, and 25% of organic farming land use [57]. Thus, considering above clauses, the perspective and priority in the scientific research should be better focused on xenobiotics that influence the greatest the food safety and security such as plant protection products, i.e., pesticides, food contact materials, i.e., plasticizers such as BPA and analogues, phthalates, and heavy metals circulating in the food chain.

## 3. Conclusions

Global policies necessitate going beyond the traditional methodologies towards cumulative risk assessment implementations for guarantying the human health through integrative actions and implementation of the One Health approach. What is more, the One Health approach has already been incorporated in the education of future consumers, assessors, managers, and scientists, and it is a key driving force in organising access to transdisciplinary science through international partnerships, joint research projects, and global conferences and social channel communications. In this sense, there is already a body of knowledge and scientific evidence to support the key role of microbiome biomarkers and its derived bioresources for contributing to modulate and intervene for improving global health. In the context of environmental health, soil is the first link-chain in the food production cycle, and afterward has a great impact on human and animal health. Thus, there is the necessity to link the environmental and microbiological research related with xenobiotics in the food chain.
